# Psychometric Characteristics of a New Scale for Measuring Self-efficacy in the Regulation of Gambling Behavior

**DOI:** 10.3389/fpsyg.2017.01025

**Published:** 2017-06-20

**Authors:** Claudio Barbaranelli, Valerio Ghezzi, Roberta Fida, Michele Vecchione

**Affiliations:** ^1^Department of Psychology, Sapienza University of RomeRome, Italy; ^2^Centro Interuniversitario per la Ricerca sulla Genesi e sullo Sviluppo delle Motivazioni Prosociali e Antisociali (CIRMPA), Sapienza Università di RomaRome, Italy; ^3^Department of Developmental and Social Psychology, Sapienza University of RomeRome, Italy; ^4^Norwich Business School, University of East AngliaNorwich, United Kingdom

**Keywords:** self-efficacy, scale development, problem gambling, validation, factor analyses, logistic regression analysis

## Abstract

Since its introduction in 1977, self-efficacy has proven to be a fundamental predictor of positive adjustment and achievement in many domains. In problem gambling studies, self-efficacy has been defined mainly as an individual's ability to avoid gambling in risky situations. The interest in this construct developed mainly with regard to treatment approaches, where abstinence from gambling is required. Very little is known, however, regarding self-efficacy as a protective factor for problem gambling. This study aims to fill this gap, proposing a new self-efficacy scale which measures not only the ability to restrain oneself from gambling but also the ability to self-regulate one's gambling behavior. Two studies were conducted in which the data from two Italian prevalence surveys on problem gambling were considered. A total of about 6,000 participants were involved. In the first study, the psychometric characteristics of this new self-efficacy scale were investigated through exploratory and confirmatory factor analyses. The results indicated the presence of two different factors: self-efficacy in self-regulating gambling behavior and self-efficacy in avoiding risky gambling behavior. The second study confirmed the replicability of the two-factor solution and displayed high correlations among these two self-efficacy dimensions and different measures of gambling activities as well as other psychological variables related to gambling (gambling beliefs, gambling motivation, risk propensity, and impulsiveness). The results of logistic regression analyses showed the particular importance of self-regulating gaming behavior in explaining problem gambling as measured by Problem Gambling Severity Index and South Oaks Gambling Screen, thus proving the role of self-efficacy as a pivotal protective factor for problem gambling.

## Introduction

This study addresses the role of self-efficacy beliefs as a protective factor for problem gambling. In particular, a new scale for measuring self-efficacy beliefs related to the regulation of one's own gambling behavior is presented. In the gambling literature, self-efficacy has been examined particularly in the context of treatment of pathological gambling, and the measures that have been developed are framed within this context, with emphasis on the ability of the patient to restrain from gambling in situations where gambling behavior is probable. However, we believe that self-efficacy is also crucial when a gambler who is not in treatment is faced with the task of regulating his or her own gaming behavior in order to not engage in excessive gambling. Moreover, a scale focused mainly on the avoidance of gambling would be of limited use in large population and epidemiological studies, where the aim is to identify those variables that may represent protective and risk factors for the development of gambling problems.

## Theoretical framework: self-efficacy within social cognitive theory

Self-efficacy represents a crucial construct within social cognitive theory (SCT, Bandura, 1986, 1997), which is focused on the acquisition of cognitive, social, emotional, and behavioral competencies as well as on the motivation and self-regulation of behavior. SCT is framed within an agentic perspective, which sees people as self-organizing, proactive, self-reflecting, and self-regulating organisms. As *agents*, people are capable of intentionally influencing their own functioning and life circumstances. Among the mechanisms of human agency considered in Bandura's theory, none is more focal or pervasive than self-efficacy beliefs (Bandura, 1991, 1997). These are individuals' beliefs regarding their ability to successfully produce given outcomes.

Self-efficacy beliefs are the basic determinants of several factors: the activities people choose; the efforts they expend in these activities; their perseverance when faced with setbacks and failures; and their causal attributions for successes and failures. Indeed, unless people believe they can produce desired results by their actions, they have little incentive to act or to persevere in the face of difficulties. Self-efficacy beliefs are dynamic factors, not general, static personality traits. They vary in magnitude depending on the difficulty of the task, their generality (some beliefs are related to specific domains while others involve a more generalized sense of mastery) and the strength with which they are held. Whatever other factors may operate as guides and motivators, they are nonetheless rooted in the core belief that one has the power to produce effects by one's actions.

Findings from different studies have demonstrated the influential role of self-efficacy beliefs in various domains of functioning (for an earlier review, see Bandura, 1997), such as learning (e.g., Pajares and Urdan, 2006), work (e.g., Judge et al., 2007), sport (Moritz et al., 2000), health and well-being (e.g., Karademas, 2006; Strobel et al., 2011), and social adjustment (e.g., Bandura et al., 2003). In these various domains, the assessment of specific self-efficacy beliefs has proven to be crucial in predicting or explaining specific behavioral outcomes (Bandura and Locke, 2003). Indeed, the specificity of self-efficacy beliefs as expressions of contextual knowledge and specific capacities has proven to be critical in studying the properties as well as the explanatory power of self-efficacy across tasks and situations. In the domain of addictive behaviors, this view is further reinforced by DiClemente et al. (1995), who argue that a measurement of self-efficacy must refer to situations that are specific to the addictive behavior considered.

## Measuring self-efficacy in the domain of gambling

Several self-efficacy scales have been developed in the domain of gambling behavior. These have been mainly focused on providing practitioners with measures to be used for the evaluation of treatment intervention aimed at reducing patients' pathological gambling. The two more commonly used scales are the Gambling Self-Efficacy Questionnaire (GSEQ) (May et al., 2003) and the Gambling Refusal Self-Efficacy Questionnaire (GRSEQ) (Casey et al., 2008).

Both scales stemmed from previous measures of self-efficacy related to alcohol addiction: the SCQ-39 (Annis and Graham, 1988) and the DRSEQ (for the GSEQ, see Young and Oei, 1996; for the GRSEQ, see Young et al., 1991). Both have been developed with consideration to the broad situational classes of factors that, according to Marlatt (1985), represent the determinants of addictive behavior and of relapse. These factors include unpleasant and pleasant emotions, physical discomfort, testing of personal control, urges and temptations, interpersonal conflicts, social pressure, and pleasant interpersonal interactions. Factor analyses indicated the presence of a single general factor for the GSEQ (see May et al., 2003; Winfree et al., 2014) or of very highly correlated factors for the GRSEQ (see Casey et al., 2008). GSEQ showed high internal consistency and high test-retest reliability (May et al., 2003); also GRSEQ showed high internal consistency for both the scales based on the 4-factor solution as well as for the overall scale derived by aggregating all the 26 items. GSEQ resulted negatively correlated with SOGS and DSM-IV indicators of problem gambling, and showed adequate reliability, sensitivity, specificity, and clinical significance: cut-off scores were developed and supported for using this measure with patients in treatment for gambling (Winfree et al., 2014). GRSEQ resulted negatively correlated with several measures of gambling problems (such as the SOGS) and with measures of distress (such as Depression, Anxiety, and Stress); it also resulted to discriminate adequately between non-problematic and problematic gamblers, as well as in pre- and post-treatment comparisons (Casey et al., 2008). Both scales have been used in rather small and non-representative samples: neither of them has been used in population studies or on representative samples of gamblers (Casey et al., 2008; Winfree et al., 2014).

GSEQ is essentially focused on the ability to control one's gambling behavior in situations that present increased odds of risky behavior (e.g., a situation in which the gambler meets a friend who suggests that they “go gambling together,” thus exposing the gambler to social pressure, or in which a person feels an urgent desire to gamble). GRSEQ is essentially focused on an individual's ability to refuse to gamble in high-risk situations (e.g., when the person is in a place where he or she usually gambles, or when the person smokes tobacco). Although the items in the scales feature different wording and response formats (GSEQ: *I would be able to control my gambling*; GRSEQ: *How confident are you that you could refuse gambling?*), they present large areas of overlap, and both are related to resisting or avoiding gambling in situations which may present increased odds of high-risk gambling behavior. None of them refer to specific agentic behaviors that allow the gambler to self-regulate his/her own gambling behavior.

## Self-regulation of gambling behaviour

As noted by Bandura (2007), “there is more to self-regulation of substance abuse, of course, than resisting pressures to consume an addictive substance” (p. 643). What Bandura says regarding substance abuse can be easily extended to problem gambling, excessive gambling, and pathological gambling. As noted above, in the domain of problem gambling research, assessment, and treatment have focused on perceived *resistance* self-efficacy. Although the ability to resist interpersonal and intrapersonal pressures to gamble is fundamental, other facets of self-efficacy may come into play in successful regulation of gambling behavior. One particularly relevant aspect is the ability to reduce harm (e.g., by restricting potential losses within gambling sessions through the exercise of controlled gambling). This refers to the ability to regulate one's own gambling behavior by acting in a way that may protect one from excessive gambling. This aspect is at the core of self-regulation mechanisms since it deals with the ability to set behavioral goals and to monitor one's own gambling behaviors. These aspects are particularly strengthened by the self-regulation, self-assessment, and self-limiting tools available in responsible gambling programs (e.g., Blaszczynski et al., 2004). Indeed, as noted by Wood and Griffiths, 2015, responsible gambling strategies aim to encourage players to restrict their gambling to a non-problematic level. In order to keep their behavior under control, players are encouraged to gain knowledge of their behavior through feedback related to their gambling, such as the amount of money they have spent and the frequency with which they play. They are also encouraged to “pre-commit” to limits on the money and the time they may spend gambling; they are urged to stick to these limits by self-excluding when the limits are reached, by taking breaks in their play, and by taking self-diagnostic tests to monitor their gambling behavior. In this regard, Wood and Griffiths (2015) recently demonstrated that *positive players* (i.e., players whose behavior and attitudes do not exhibit problems or elicit concerns with regard to their gambling) adopt the following as personal strategies for responsible gambling: setting spending limits before playing, evaluating how much they can afford to lose before playing and setting time limits for playing. As noted by the authors, “these strategies are associated with a positive play experience” (Wood and Griffiths, 2015, p. 1,729), while the absence of these strategies is significantly associated with problematic gambling. In this regard, these players' proactive stances are consistent with the stress placed by Blaszczynski et al. (2004) on individuals' personal responsibility in their level of gambling participation as a basic tenet of responsible gambling.

From these premises and considerations, a new scale for measuring self-efficacy related to gambling behavior has been developed, and its characteristics are presented in this paper. The scale aims to overcome the limits of both GSEQ and GRSEQ by making available an instrument focused not only on avoidance and refusal to gamble in risky situations but also on the proactive self-regulation of gaming behavior conducive to positive play and, thus, to the prevention of excessive gambling. This instrument would, as a result, be more suited for use in large population studies on gamblers not involved in psychological treatment. In this paper, we present two studies that are focused on the psychometric characteristics of the scale as far as the internal structure of the items is concerned, the correlation with other measures related to gambling behaviors and the impact of self-efficacy as a protective factor for problem gambling.

## Study 1: psychometric characteristics of the multidimensional gambling self-efficacy scale

The main aim of the first study was to examine the factorial structure and reliability of the Multidimensional Gambling Self-Efficacy Scale (MGSES)—a new scale for measuring gambling self-efficacy—in two large and representative samples of gamblers. First, we will provide details about the process used to develop the initial pool of items included in the scale. Second, we will present the results of the dimensionality and reliability/internal consistency analyses.

### Method

#### Participants

This study considered two independent samples: (a) an *overall* sample of players reflecting the overall population of gamblers who gambled at least once in the 12 months before the data collection at any game involving money, without any reference to the type of game played to be used as an inclusion criterion; (b) a smaller sample of players who, in the 12 months before the data collection, gambled at least once at any *online* game involving money.

Each sample was representative of their respective populations of *overall* and *online* adult Italian gamblers (18 years or older). The *overall* sample consisted of 2,015 participants, 54% of whom were males. Their ages ranged from 18 to 87 years (*M* = 47.43, *SD* = 15.43). In terms of education, 18% had not continued past elementary studies; 40% had stopped at primary studies, 33% had stopped at secondary studies; and 9% had stopped at university studies. Of the participants, 25% were single, 64% were married and 11% were separated or widowed. Their modal occupations included employers/office workers (16%), manual workers (19%), and retirees (20%). The *online* sample consisted of 1,005 participants, 67% of whom were male. Their ages ranged from 18 to 64 years (*M* = 37.55, *SD* = 12.42). Of these, 1% had undergone only elementary studies; 12% had stopped at primary studies; 57% had stopped at secondary studies; and 30% had stopped at university studies. In terms of marriage status, 45% were single, 51% were married and 5% were separated or widowed. Their modal occupations were included employers/office workers (32%), manual workers (10%), and students (14%).

Regarding the gambling behavior exhibited in the last 12 months, participants in the *overall* sample played an average of about three different games (*M* = 3.5, *SD* = 2.44). The most played games were instant lotteries (84%) and lotto/other lotteries (81%); betting was played by about 17%, slots/VLT by about 12%, bingo as well as online games by about 11%, games at casinos by about 2%. Seventy-eight percentage of participants dedicated less than 30 min per day to gambling, while participants who gambled for 2 h or more per day were 4%. Participants whose maximum daily expense for gambling was less than 20 Euros were 86%, while those whose maximum daily expense for gambling was higher than 100 Euros were 3%. About 4% of participant had one or both parents who are or used to be excessive gamblers. Participants in the *online* sample played an average of about 11 different games (*M* = 10.79, *SD* = 6.6). All participants played online, since having played online at least once was the criterion for inclusion in the research. Considering games played online, the most played games were betting (63%), poker (55%), lotto (45%), casino games (40%). Considering games not played online, the most played games were lotto/other lotteries (86%), instant lotteries (79%), and betting (70%); slots/VLT were played by about 41%, bingo by about 40%, games at casinos by about 26%. Twenty-five percentage of participants dedicated less than 30 min per day to gambling, while participants who gambled for 2 h or more per day were 17%. Participants whose maximum daily expense for gambling was less than 20 Euros were 70%, while those whose maximum daily expense for gambling was higher than 100 Euros were 7%.About 15% of participant had one or both parents who are or used to be excessive gamblers. These different patterns of gambling behaviors further confirm the diversity of the two samples considered.

#### Procedure

Data were collected by Ipsos, one of the leading market research organizations operating in Italy (http://www.ipsos.it/), in October and November 2012 within a national study on the prevalence of problem gambling, and on risk and protective factors for problem gambling in Italy. The target number of subject (2,000 and 1,000 respectively in the *overall* and in the *online* samples) was defined in order to have a standard error of the 95% confidence interval for prevalence estimates of 1% in the *overall* sample and of 2% in the *online* sample. A quota sampling strategy, balanced by geographical area (including four areas), city size (including five groups), and age/gender (including 12 groups), was used. For the *overall* sample, participants were contacted by an interviewer and invited to fill out a questionnaire of about 300 items. The questionnaire was individually administered to participants in their homes. For the *online* sample, participants were administered a questionnaire of about 250 items using the Computer Assisted Web Interviewing (CAWI) methodology. Persons who initially agreed to participate but later declined were replaced by other participants with homogeneous characteristics. Individuals received a fee of about 20 Euros for their participation. After data collection was complete, participants were weighted in order to maximize the sample's representativeness of the target population. The ethic Committee of CIRMPA—Sapienza University of Rome approved the research. Ethical procedures concerning privacy, anonymity and confidential treatment of data were respected: an informed consent sheet was signed by all participants before the questionnaire and interview were administered. All participants were allowed to leave the study at any time. All procedures were performed in accordance with the ethical standards of the institutional and/or national research committee.^1^

#### Materials

The questionnaire administered comprised different scales. In particular, a first set of variables measured gambling behavior (number of games played, types of games, etc.), a second set comprised indicators of problem gambling (PGSI and SOGS), a third set comprised possible risk factors (e.g., beliefs about gambling, motivation to gamble, etc.) and protective factors (e.g., self-efficacy, life-satisfaction, etc.) for problem gambling, a fourth set comprised variables that might represent other possible sources of risk or of comorbidity for problem gambling (e.g., impulsiveness, risk propensity, depression, life events, psychological distress, etc.). A final set comprised variables related to respondents' perception of the problem gambling phenomenon.

Among the scales administered in the survey, we considered in this first study only the MGSES. As noted in the introduction, this new self-efficacy scale was developed to overcome the limitations of the previous measures of gambling self-efficacy. Following a “top-down” approach the items were generated after an inspection of the scientific literature regarding self-regulatory processes related to problem gambling, and self-regulation, self-assessment, and self-limiting processes stressed within responsible gambling tools and programs. Two sets of items were then developed with the aim of defining two different self-efficacy subscales. The first set was comprised of items that were essentially focused on the avoidance of gambling in situations that (according to the examined literature) exposed gamblers to a risk of excessive gambling. These situations resulted substantially consistent with those considered in other scales aimed at assessing self-efficacy in the domain of gambling. The self-efficacy in avoiding gambling behavior scale was then assembled. It was comprised of 11 items assessing the degree to which players perceive themselves to be capable of avoiding gambling in the following circumstances: (a) when under stress or when experiencing negative affects/states; (b) during leisure time; (c) when in social situations; (d) when in conflict situations; and (e) when feeling the urge to play. A second set was comprised of items essentially focused on reducing the probability of harmful outcomes of gambling through the exercise of controlled gambling. These items refer to the ability to regulate one's own gambling by acting in a way that may protect oneself from engaging in excessive gambling. The items content was based upon those responsible gambling strategies aimed at encouraging players to restrict their gambling to a non-problematic level and to pursue a “positive playing” (see Wood and Griffiths, 2015). The self-efficacy in self-regulating gambling behavior scale was then assembled. It was comprised of six items assessing the degree to which a gambler was capable of the following: (a) spending only the amount of money initially decided upon; (b) ceasing play when a pre-decided time limit was reached; (c) avoiding spending in gambling the change or money that must be spent to buy other things; (d) sticking to one's decision not to play, despite temptation; and (e) stop playing to win back (“chasing” behavior). The formulation of the items was discussed with three experts working since at least 5 years in the field of responsible gambling programs (one was a psychologist and the other two were graduated in other disciplines). The feedback of the three experts was used as a check for the completeness of the situations and of the behaviors examined in the scale. Experts' opinions helped furthermore in the clarification of the item wording (see Schuman and Presser, 1996). All 17 items of the two subscales, as well as the complete five-step response formats (from 1 = not at all, to 5 = completely) are reported in the Appendix in Supplementary Materials.

### Data analysis

Items were first evaluated in terms of descriptive statistics and univariate normality. Then, a cross-validation procedure (Byrne, 2010) was applied to assess the factorial structure of MGSES. Specifically, an exploratory factor analysis (EFA) was conducted, first on the *overall* sample. In this EFA, the number of factors to retain was identified by means of a parallel analysis (see Hayton et al., 2004) comparing the real data eigenvalues with those derived from random artificially generated data, maintaining the same sample size and number of observed indicators. The factors to be retained were determined by the number of “real data” eigenvalues that were higher than the corresponding number derived from random datasets. Fit indices of the final EFA model were also computed. The fit of the final EFA solution was compared, by means of a chi-square difference test, to that of a solution with the same number of factors suggested by parallel analysis, minus one. Geomin factor rotation was used for the EFA model (see Muthén and Muthén, 1998-2016). Once the final factorial solutions were calibrated with EFA on the *overall* sample, a confirmatory factor analysis (CFA) was performed to cross-validate the factorial structure on the *online* sample. Since the two samples present substantial differences with respect to demographic characteristics, pattern of gambling, inclusion criteria and method of assessment, we believe these differences would substantially reduce the probability to obtain replicable results only by chance, and enhance the value of the replicable results obtained. The overall model fit was evaluated using a multifaceted approach including the following (Kline, 2016): (i) chi-square test; (ii) Root Mean Square Error of Approximation (RMSEA, Steiger, 1990; MacCallum et al., 1996; if ≤ 0.08, the model shows a good fit); iii) Comparative Fit Index (CFI, Bentler, 1990; if ≥ 0.90, the model shows an acceptable fit); and (iv) Standardized Root Mean Squared Residual (SRMR, Hu and Bentler, 1999; if ≤ 0.08, the model shows an acceptable fit). The reliability of MGSES dimensions was evaluated in both samples in terms of internal consistency with the Cronbach's alpha coefficient.

### Results

#### Descriptive statistics

Table 1 shows a summary of descriptive of MGSES items in both the *overall* and *online* samples. Skewness and kurtosis values (especially in the *overall* sample) suggest that the distributions of items do not perfectly fit univariate normality assumptions. Specifically, items mainly present negatively skewed distributions (Tabachnick and Fidell, 2007). These will be taken into account for further analyses using robust estimators in order to deal with these departures from univariate normality.

**Table 1:** Summary descriptive Statistics of MGSES Items in *overall* and *online* Samples.

	***Overall* sample (*N* = 2,015)**				***Online* sample (*N* = 1,005)**			
	**Min**	**Max**	***M***	***SD***	**Min**	**Max**	***M***	***SD***
*M*	4.05	4.51	4.23	0.16	3.53	4.06	3.77	0.15
*SD*	0.82	1.15	1.01	0.11	1.04	1.19	1.12	0.05
Skewness	−1.90	−0.88	−1.26	0.30	−0.79	−0.24	−0.54	0.16
Kurtosis	−0.27	3.52	0.91	1.08	−0.89	−0.08	−0.56	0.22

*M, Mean; SD, Standard Deviation*.

#### Factorial structure and reliability of MGSES

Given the distribution of MGSES items in both samples, EFA and CFA were carried out using Robust Maximum Likelihood estimators (MLR in Mplus software; see Muthén and Muthén, 1998-2016). The first three real data eigenvalues were 10.62, 1.75, and 0.76, while the first three eigenvalues associated with artificial data (based on 1,000 replications) were 1.16, 1.13, and 1.10. Therefore, parallel analysis suggests the presence of two factors. Moreover, the model comparison of the two-factor EFA model with a one-factor model suggests an improvement in model fit, $\Delta \chi_{\left(\Delta \text{df}=16\right)}^{2}$ = 1,995.998 and *p* < 0.001. Thus, two factors were retained for the final EFA solution. The overall model fit was satisfying: $\chi_{\left(df=103\right)}^{2}$ = 1,049.49, *p* < 0.001, RMSEA = 0.068, CFI = 0.932, and SRMR = 0.029. In the left part of Table 2, factor loadings of the final Geomin oblique solution are presented. Consistent with the theoretical dimensions hypothesized when developing MGSES items, the two factors are clearly interpretable as *self-efficacy in self-regulating gambling behavior* (REG_SE) and *self-efficacy in avoiding gambling behavior* (AV_SE) since the final solution has a very simple structure in both samples, being all cross loadings lower than |0.20|. The correlation between factors was 0.69 (*p* < 0. 05).

**Table 2:** Standardized factor loadings on both samples for the final two-factor EFA solution and from the CFA model.

	**EFA—*Overall* sample (*N* = 2,015)**		**CFA—*Online* sample (*N* = 1,005)**	
	**REG_SE**	**AV_SE**	**REG_SE**	**AV_SE**
it1	**0.89**	−0.03	0.87	
it2	**0.88**	0.01	0.87	
it3	**0.89**	−0.05	0.88	
it4	**0.82**	0.06	0.87	
it5	**0.57**	0.19	0.78	
it6	**0.73**	0.16	0.87	
it7	0.13	**0.74**		0.84
it8	0.07	**0.81**		0.87
it9	−0.02	**0.76**		0.76
it10	0.03	**0.84**		0.81
it11	0.02	**0.80**		0.77
it12	0.00	**0.75**		0.75
it13	−0.05	**0.91**		0.83
it14	−0.05	**0.91**		0.87
it15	−0.03	**0.81**		0.73
it16	0.01	**0.84**		0.83
it17	0.01	**0.84**		0.84

*Principal factor loadings are presented in bold for the EFA solution. REG_SE, Self-efficacy in self-regulating gaming behavior; AV_SE, Self-efficacy in avoiding gambling behavior*.

Once the EFA final solution was established, the factorial structure was replicated by means of CFA on the *online* sample. In terms of goodness of fit, the results were satisfying: $\chi_{\left(df=118\right)}^{2}$ = 1,583.26, *p* < 0.001, RMSEA = 0.079, CFI = 0.916, and SRMR = 0.033. Factor loadings are presented in the right part of Table 2, and they were all higher than 0.70. The correlation of latent factors was 0.78 (*p* < 0.001). As a check of the discriminant validity of the factors, a CFA model with a single factor was examined and then compared with the two-factor model. Results indicated that the single-factor model's fit with the data was much poorer than that of the two-factor model, with a $\Delta \chi_{\left(\Delta \text{df}=1\right)}^{2}$ = 1,544.19 and *p* < 0.001. Cronbach's alphas in the *overall* sample were 0.93 and 0.96 respectively for REG_SE and for AV_SE, while in the *online* sample they were 0.94 and 0.96. respectively for REG_SE and for AV_SE.

### Discussion

Support for the structural validity and reliability of the MGSES is fully achieved in the results of this first study. Factor analysis showed that the 17 items clearly measure the two hypothesized dimensions of self-regulating and avoiding self-efficacy. Indeed, all of the items were good indicators of the intended factor, and the psychometric properties of these two scales were excellent. This was proven by the clear, simple factorial structure of solutions when examined by means of EFA, where all cross-loadings were negligible; the simple structure was further replicated through the thorough tests of CFA on a sample whose characteristics are rather different from those of the sample used for EFA, and this enhances the value of the replicable results (American Psychological Society, 2015). High Cronbach's alpha coefficients attested the internal coherence of the scales. We have to acknowledge that in order to completely establish reliability estimates, a test-retest coefficient had to be derived. Unfortunately, the design of the study did not allow to test for this type of reliability estimate. Finally, the discriminant validity of the two factors was supported by the poor fit reached by an alternative model in which a unique factor was posited. While the factor correlation indicated a solid stem common to the two dimensions, these two dimension were significantly different, thus representing two important facets or aspects of self-efficacy related to gambling behavior. Overall, the findings from this study provided evidence of the quality of the MGSES. Further investigations of the stability of this factorial structure and of the validity of the MGSES are presented in the following study.

## Study 2: factorial solution replicability, discriminant, and criterion validity of the MGSES

With this second study, we aimed to test both the replicability of the MGSES factor structure on two independent samples and its measurement invariance across different samples. Moreover, we aimed to test the criterion validity of the scale by examining the following: (a) the correlation of the two specific SE dimensions on relevant criteria/variables, including problem gambling measures, gambling behaviors, gambling beliefs, gambling motivation, and risk propensity; (b) the unique contribution of self-efficacy dimensions in explaining problem gambling by means of logistic regression analyses.

### Method

#### Participants

This study, like the previous one, considered two different and independent samples: (a) an *overall* sample of players reflecting the overall population of gamblers who gambled at least once in the 12 months before the data collection at any game involving money, without any reference to the type of game played to be used as an inclusion criterion; (b) a smaller sample of players who, in the 12 months before the data collection, gambled at least once at any *online* game involving money. As in study 1, the samples were representative of their respective populations of adult Italian gamblers. The *overall* sample consisted of 2,030 participants, 58% of whom were males. Their ages ranged from 18 to 87 years (*M* = 48, *SD* = 16). In terms of education, 17% had not continued past elementary studies; 43% had stopped at primary studies; 32% had stopped at secondary studies; and 8% had stopped at university studies. Of the participants, 26% were single, 61% were married and 13% were separated or widowed. Their modal occupations included employers/office workers (13%), manual workers (22%), housewives (14%), and retirees (20%). The *online* sample consisted of 1,000 participants, 70% of whom were males. Their ages ranged from 18 to 64 years (*M* = 37.8, *SD* = 12.5). Of the participants, 1% had not continued beyond elementary studies; 15% had stopped at primary studies; 57% had stopped at secondary studies; and 27% had stopped at university studies. In terms of marital status, 47% were single, 48% were married, and 5% were separated or widowed. Their modal occupations included employers/office workers (31%), manual workers (12%), and housewives (13%).

Regarding the gambling behavior exhibited in the last 12 months, participants in the *overall* sample played an average of about three different games (*M* = 3.1, *SD* = 2.33). The most played games were instant lotteries (82%) and lotto/other lotteries (73%); betting was played by about 14%, slots/VLT by about 13%, online games by about 12%, bingo by about 8%, games at casinos by about 2%. Seventy-four percentage of participants dedicated less than 30 min per day to gambling, while participants who gambled for 2 h or more per day were 6%. Participants whose maximum daily expense for gambling was less than 20 Euros were 83%, while those whose maximum daily expense for gambling was higher than 100 Euros were 1%. About 3% of participant has one or both parents who are or used to be excessive gamblers. Participants in the *online* sample played an average of about 11 different games (*M* = 11.20, *SD* = 8.4). As in study 1, all participants played online, since having played online at least once was the criterion for inclusion in the research. Considering games played online, the most played games were betting (62%), lotto (53%), and poker (44%). Considering games not played online, the most played games were lotto/other lotteries (77%), instant lotteries (73%), and betting (55%); slots/VLT were played by about 37%, bingo by about 37%, games at casinos by about 32%. Thirty-five percentage of participants dedicated less than 30 min per day to gambling, while participants who gambled for 2 h or more per day were 15%. Participants whose maximum daily expense for gambling was less than 20 Euros were 52%, while those whose maximum daily expense for gambling was higher than 100 Euros were 7%. About 12% of participant has one or both parents who are or used to be excessive gamblers. As in the case of study 1, these different patterns of gambling behaviors further confirm the diversity of the two samples considered.

#### Procedure

Data were collected by IPSOS in October and November 2014. The same procedures for sampling strategy, questionnaire administration, data collection and ethical issues used in study 1 were used also in study 2: we refer to study 1 for a detailed description.^2^

#### Materials

The questionnaire administered for study 2 comprised substantially the same sets of variables described in the methods section of study 1, to which we refer for a more detailed description. In particular, in study 2 we focused our attention on the following scales:

*Multidimensional Gambling Self-Efficacy Scale* (MGSES) described in Study 1. Factorial structure and reliability indices will be described in the results section, along with the measurement invariance tests.

*Measures of Gambling Behaviors include* gambling frequency, time spent gambling, the maximum amount of money spent gambling in a single day, the number of games played, the type of games played, and the familiarity (i.e., the presence in the gambler family) of the gambling problems.

*Problem Gambling* was measured by the Italian versions of SOGS and of PGSI (Barbaranelli et al., 2013). SOGS is a dichotomous 20-item scale that evaluates the presence of problem gambling (Lesieur and Blume, 1987). PGSI, another scale that measures problem gambling, uses 9 items that each have four response options, from 0 = never to 3 = almost always (Ferris and Wynne, 2001). The reliability of SOGS was 0.84 and 0.89, and the reliability of PGSI was 0.92 and 0.96, respectively, in the *overall* and *online* samples.

*Erroneous Gambling Beliefs* were assessed with 10 items from the Gamblers' Beliefs Questionnaire (Steenbergh et al., 2002). In particular, the items measured gamblers' erroneous beliefs: their overestimation of their control over the outcomes of games and, thus, their chances of winning (the illusion of control; e.g., *My knowledge and skill in gambling contribute to the likelihood that I will make money*) and their belief in the probability of a win if they continue to gamble (perseverance; e.g., *When I am gambling, “near misses”—moments when I almost win—remind me that, if I keep playing, I will win*). Previous factor analyses demonstrated the presence of a single factor underlying the 10 items. The reliability of the scale was 0.94 in both *overall* and *online* samples.

*Gambling Motivations* were assessed using 12 items adapted from the Motives for Gamble scale (Cotte, 1997; Rousseau and Venter, 2002). In particular, the items measured gamblers' symbolic motives (e.g., *Gambling is a way to show others that I am good*), economic motives (e.g., *Gambling is a good way to earn money*), and hedonic motives (e.g., *Gambling is an exciting pastime*). Previous factor analyses indicated the presence of three correlated factors underlying the 12 items. Reliability coefficients of the three scales in *overall* and *online* samples, respectively, were 0.90 and 0.86 for symbolic motives, 0.91 and 0.74 for hedonic motives and 0.87 and 0.91 for economic motives.

*Risk taking* was assessed with 11 items from the Stimulating Risk Taking scale (Zaleskiewicz, 2001) and from the Declared Risk Taking scale (Dahlbäck, 1990). These items measure an individual's propensity to take risks as a way of providing stimulation, excitement and arousal; (e.g., *Every time I take a risk, I experience a pleasant feeling of excitement*) and of declare the benefit to oneself of more often engaging in risky behaviors (e.g., *I think I am often less wary of other people*). Previous factor analyses evidenced the presence of a single factor underlying the 11 items. The reliability of the scale was 0.95 in both *overall* and *online* samples.

*Impulsiveness* was assessed with four items from the Self-Control scale (Tangney et al., 2004) and four items from Barratt Impulsiveness Scale (BIS, Patton et al., 1995). Self-control refers to “the ability to override or change one's inner responses, as well as to interrupt undesired behavioral tendencies and refrain from acting on them” (Tangney et al., 2004, p. 275; e.g., *Sometimes I cannot stop doing something, even though I know it is wrong*). BIS measures the personality trait of impulsiveness. In particular, we considered four items from the BIS Motor Impulsiveness subscale, which assesses the tendency to act on the spur of the moment and the consistency of one's lifestyle (e.g., *I act on the spur of the moment)*. Previous factor analyses indicated the presence of a single factor underlying the eight items. The reliability of the scale was 0.84 in the *overall* sample and 0.87 in the *online* sample.

All scale items were rated with five response options (from 1 = doesn't describe me at all, to 5 = describes me very much), with the exception of MGSES, SOGS, and PGSI.

#### Data analysis

The replicability of the two-factor structure of MGSES was evaluated by means of CFA in both samples, and these models were evaluated following the same fit criteria used in Study 1. Furthermore, measurement invariance (Meredith, 1993) of MGSES was tested by comparing series of nested multigroup confirmatory factor models (MG-CFA) ordered in terms of increasing complexity, considering separately: (a) *overall* vs. *online* samples, and (b) males vs. females. Specifically, for each of the two invariance analyses we first ran the two-factor CFA model simultaneously on two groups without imposing constraints on model parameters (this is called *configural invariance* model). In the second nested model, factor loadings were constrained to equality across groups (this is called *metric invariance* model), while in the third model, equality constraints were also applied on item intercepts (this is called *scalar invariance* model). Finally, the equality of residual variances was added to the scalar model (this is called *strict invariance* model). To evaluate whether constraints were tenable, statistical comparison among each adjacent couple of models was performed by means of ΔCFI (Cheung and Rensvold, 2002). If ΔCFI across adjacent models was < 0.01, the more parsimonious model could not be rejected.

The criterion validity of MGSES was assessed by zero-order correlations of its two dimensions with the aforementioned scales related to gambling (e.g., SOGS, PGSI, etc.) and with typical behavioral indicators of gambling (e.g., the number of games played in the last 3 months, average time per day spent playing, etc.). Due to the correlation between the two MGSES factors, partial correlations were also computed in order to better evaluate the association of each MGSES factor with the various variables considered, controlling for the other MGSES factor. Finally, hierarchical logistic regression was used to evaluate the unique contribution of MGSES dimensions above and beyond demographics and other relevant gambling-related variables in explaining problem gambling as a criterion variable obtained from a combined use of SOGS and PGSI (see Barbaranelli et al., 2013).

### Results

#### Replicability of the MGSES factorial structure

For the *overall* sample, the fit was satisfying: $\chi_{\left(df=118\right)}^{2}$ = 1,127.45, *p* < 0.001, RMSEA = 0.065, CFI = 0.938, and SRMR = 0.028. The latent correlation among the two MGSES factors was 0.76 (*p* < 0.001), and αs were, respectively, 0.94 for REG_SE and 0.96 for AV_SE. Also, for the *online* sample, the fit was satisfying: $\chi_{\left(df=118\right)}^{2}$ = 696.95, *p* < 0.001, RMSEA = 0.070, CFI = 0.938, and SRMR = 0.029. Latent correlation among the two MGSES factors was 0.81 (*p* < 0.001) and alphas were, respectively, 0.94 for REG_SE and 0.96 for AV_SE. The factorial structure of MGSES derived from Study 1 closely fits the data of both samples considered for Study 2. Factor loadings for both samples are reported in Table 3. They are very high and are similar to those found in the Study 1 samples. As for Study 1, the two-factor model was compared with a single-factor model in order to evaluate the discriminate validity of MGSES dimensions. In both the *overall* and the *online* samples, the single-factor model produced a significantly inferior model fit, with a $\Delta \chi_{\left(\Delta \text{df}=1\right)}^{2}$ = 1,685.07, *p* < 0.001 and a $\Delta \chi_{\left(\Delta \text{df}=1\right)}^{2}$ = 569.23, *p* < 0.001, respectively.

**Table 3:** Standardized factor loadings on both samples for the CFA model.

	***Overall* sample (N = 2,030)**		***Online* sample (N = 1,000)**	
	**REG_SE**	**AV_SE**	**REG_SE**	**AV_SE**
it1	0.85		0.88	
it2	0.89		0.88	
it3	0.86		0.87	
it4	0.89		0.89	
it5	0.76		0.80	
it6	0.88		0.89	
it7		0.86		0.89
it8		0.89		0.89
it9		0.73		0.81
it10		0.86		0.84
it11		0.83		0.86
it12		0.74		0.82
it13		0.88		0.83
it14		0.89		0.88
it15		0.78		0.79
it16		0.86		0.85
it17		0.86		0.88

*REG_SE, Self-efficacy in self-regulating gaming behavior; AV_SE, Self-efficacy in avoiding gambling behavior*.

#### Measurement invariance of MGSES

Table 4 shows the results of the two measurement invariance tests performed respectively across samples and gender. First, the configural models adequately fit the data. When introducing constraints on factor loadings, the requirements for metric invariance tenability were met in both tests (i.e., gambling sample—*overall* vs. *online*—and gender). Then, multigroup constraints were set on item intercepts. These constraints did not significantly worsen models' fit, so the conditions of measurement scalar invariance were also satisfied for both tested cases. Finally, results from model for strict invariance suggest that constraints on residual variances of items were also tenable. In sum, MGSES reached the full strict invariance across the two testing conditions that were considered. This result is an important prerequisite not only for studying differences at the latent level between samples but also for making meaningful comparisons on the level of observed scores (DeShon, 2004). In this case, such a comparison would make sense both when considering different samples of gamblers (*overall* vs. *online*) and when focusing on gender differences (males vs. females).

**Table 4:** Goodness of fit indices of CFA models for measurement invariance

**Model**	**χ^2^**	**df**	**RMSEA**	**CFI**	**SRMR**	**ΔCFI**
**CROSS-SAMPLE INVARIANCE**						
*Overall* sample (*n* = 2,030)	1,127.45	118	0.065	0.938	0.030	−
*Online* Sample (*n* = 1,000)	696.95	118	0.070	0.928	0.029	−
Configural	1,838.90	236	0.067	0.938	0.030	−
Metric	1,960.97	251	0.067	0.934	0.043	0.004
Scalar	2,053.62	266	0.067	0.931	0.046	0.003
Strict	2,268.07	283	0.068	0.923	0.059	0.008
**GENDER INVARIANCE**						
Males (*n* = 1,767)	1,089.51	118	0.068	0.939	0.029	−
Females (*n* = 1,232)	733,41	118	0.064	0.942	0.028	−
Configural	1,800.84	236	0.066	0.940	0.029	−
Metric	1,859.70	251	0.065	0.938	0.031	0.002
Scalar	1,926.50	266	0.064	0.936	0.031	0.002
Strict	1,966.38	283	0.063	0.935	0.035	0.001

*Results are based on MG-CFA models performed over the four available samples (Overall and Online samples of Study 1 and Study 2)*.

#### Criterion validity

Table 5 reports correlations of MGSES dimensions with other gambling-related measures. Correlations were all negative and statistically significant. These correlations were higher in the *overall* sample than in the *online* sample. Remarkably, both factors of MGSES were strongly and negatively associated with problem gambling measures. As noted above, since the two MGSEG factors were highly correlated, we computed partial correlation in order to measure the unique association of MGSES factors to the variables considered. As can be seen in Table 5, partial correlations clearly demonstrated that the association of REG_SE with all variables, although decreasing when controlling for AV_SE, remained high and significant in both samples. In its turn, AV_SE displayed a strong reduction in its association with all variables. However, its partial correlations remained significant among almost all variables especially in the *overall* sample. This indicates the added value of AV_SE with respect to the variance already accounted for by REG_SE. Remarkably, REG_SE was indicated to be the more important aspect of self-efficacy in that it was associated with the two measures of problem gambling, while AV_SE association was rather marginal.

**Table 5:** Zero-order and partial correlations of MGSES dimensions with other scales related to gambling.

	**SOGS**	**PGSI**	**ERR_BEL**	**SYMB_M**	**ECON_M**	**HEDO_M**	**RISK**	**IMPULS**
***OVERALL* SAMPLE (*N* = 2,030)**								
**Zero order correlations**								
REG_SE	−0.55	−0.58	−0.56	−0.49	−0.38	−0.49	−0.54	−0.37
AV_SE	−0.43	−0.47	−0.54	−0.45	−0.35	−0.47	−0.47	−0.32
**Partial correlations**								
REG_SE	−0.39	−0.40	−0.27	−0.26	−0.19	−0.24	−0.33	−0.21
AV_SE	−*0.03*	−0.08	−0.23	−0.15	−0.11	−0.18	−0.12	−0.07
***ONLINE* SAMPLE (*N* = 1,000)**								
**Zero order correlations**								
REG_SE	−0.38	−0.43	−0.34	−0.36	−0.26	−0.21	−0.29	−0.21
AV_SE	−0.27	−0.32	−0.32	−0.31	−0.20	−0.23	−0.31	−0.19
**Partial correlations**								
REG_SE	−0.27	−0.31	−0.16	−0.20	−0.17	−*0.04*	−0.08	−0.10
AV_SE	*0.02*	*0.03*	−0.10	−0*.05*	−*0.00*	−0.12	−0.15	−*0.05*

*REG_SE, Self-efficacy in self-regulating gaming behavior; AV_SE, Self-efficacy in avoiding gambling behavior; SOGS, South Oaks Gambling Screen total score; PGSI, Problem Gambling Severity Index total score; ERR_BEL, Gamblers erroneous beliefs; SYMB_M, Symbolic motives for gambling; ECON_M, Economic motives for gambling; HEDO_M, Hedonic motives for gambling; RISK, Risk Taking; IMPULS, Impulsiveness. Correlations were significant for p < 0.05, excepting those reported in italics. Partial correlation for REG_SE were computed controlling for AV_SE; Partial correlation for AV_SE were computed controlling for REG_SE*.

Table 6 shows correlations among MGSES factors and some typical measures of gambling behavior. With regard to the number of games played in the last 12 or 3 months, similar correlations were detected with both MGSES dimensions across the two samples, with correlations in the *overall* sample generally higher than those in the *online* sample. The variables resulting in a higher correlation with MGSES were the number of games played, playing SLOTS/VLT, the maximum amount of money spent per day and the average time spent playing. We again obtained partial correlations for a better understanding of the unique association among MGSES factors and the various measures of gambling behavior. Notably, while the contribution of REG_SE almost always remained significant after controlling for AV_SE, the contribution of AV_SE disappeared when controlling for REG_SE with only a few exceptions and only in the *overall* sample. Again, REG_SE appears to be the crucial aspect of self-regulation associated with excessive gambling.

**Table 6:** Zero-order correlations and partial correlations (within parentheses) of MGSES dimensions with measures of gambling behaviors.

	***Overall* sample (*N* = 2,030)**		***Online* sample (*N* = 1,000)**	
**Gambling behavior**	**REG_SE**	**AV_SE**	**REG_SE**	**AV_SE**
Number of games played (past 12 months)	−0.26^***^ (−0.11^***^)	−0.26^***^ (−0.10^***^)	−0.28^***^ (−0.16^***^)	−0.22^***^(−0.03^ns^)
Number of games played (past 3 months)	−0.30^***^ (-0.09^***^)	−0.28^***^ (-0.09^***^)	−0.22^***^(-0.11^***^)	−0.19^***^(−0.04^ns^)
*Single Games*				
- Lotteries	−0.04^ns^ (0.05^*^)	−0.08^***^ (−0.09^***^)	−0.19^***^(−0.10^**^)	−0.16^***^(−0.03^ns^)
- Instant lottery	0.03^ns^ (0.04^ns^)	0.00^ns^ (-0.03^ns^)	0.05^ns^(0.03^ns^)	0.03^ns^(−0.01^ns^)
- Bingo	−0.12^***^ (−0.05^*^)	−0.12^***^ (−0.04^ns^)	−0.14^***^(−0.09^**^)	−0.12^***^(−0.01^ns^)
- Betting	−0.17^***^ (−0.08^***^)	−0.15^***^ (-0.05^*^)	−0.18^***^(−0.12^***^)	−0.13^***^(0.01^ns^)
- Slots/VLT	−0.30^***^ (−0.23^***^)	−0.20^***^ (0.03^ns^)	−0.27^***^(−0.18^***^)	−0.21^***^(−0.01^ns^)
- Playing in casinos	−0.11^***^ (−0.06^***^)	−0.10^***^ (−0.03^ns^)	−0.21^***^(−0.10^**^)	−0.19^***^(−0.05^ns^)
Amount of money spent in a single day	−0.40^***^ (−0.27^***^)	−0.32^***^ (−0.03^ns^)	−0.26^***^(−0.16^***^)	−0.21^***^(−0.01^ns^)
Average time per day spent playing	−0.41^***^ (−0.26^***^)	−0.33^***^ (−0.03^ns^)	−0.26^***^(−0.14^***^)	−0.22^***^(−0.04^ns^)
Having one or both parents who are or used to be excessive gamblers	−0.13^***^ (−0.09^***^)	−0.09^***^ (0.01^ns^)	−0.13^***^(-0.09^**^)	−0.10^***^(0.00^ns^)

RE_SE, Self-efficacy in self-regulating gaming behavior; AV_SE, Self-efficacy in avoiding gambling behavior; ns, statistically non-significant;

* p < 0.05;

** p < 0.01 and

*** *p < 0.001. Partial correlation for REG_SE were computed controlling for AV_SE; Partial correlation for AV_SE were computed controlling for REG_SE*.

As a final step of criterion validity, two hierarchical logistic regressions (one per sample) were carried out in order to explain problem gambling defined on the basis of a combined use of SOGS and PGSI criteria. In Step 1, background variables (i.e., gender, age, education level, and income) were added to the regression model; in Step 2, gambling-related measures (including MGSES dimensions) were included. For sake of clarity in the results interpretation, REG_SE and AV_SE were re-coded so that higher scores reflected a higher lack of self-efficacy. The results are presented in Table 7. In both samples, background variables did not account for problem gambling, while the introduction of variables related to gambling in Step 2 significantly increased the explained variance of problem gambling. Nagelkerke's R^2^ indicated, in both samples, a robust association between explanatory variables and the criterion in Step 2, and the increase in explanation was significantly high in both samples. In both samples, familiarity was the variable with the highest association with problem gambling, and REG_SE was the second most important variable. While in the *overall* sample, hedonic motivation was almost comparable to REG_SE in terms of explanatory magnitude (as captured by the odds ratio), in the *online* sample, none of the significant variables other than familiarity had a comparable relative importance with respect to REG_SE. This result further confirms the importance of this aspect of self-efficacy as a protective factor with respect to problem gambling. From a practical point of view, the result related to REG_SE means that the probability of finding a gambler lacking in self-efficacy is almost four times higher among problem gamblers than among non-problem gamblers.

**Table 7:** Hierarchical logistic regression results for the land-based and online samples.

	***Overall* sample (*N* = 2,030)**			***Online* sample (*N* = 1,000)**		
	**B**	**SE**	**OR**	**B**	**SE**	**OR**
**BACKGROUND VARIABLES (STEP 1)**						
Gender (base = male)	0.03	0.30	1.03	−0.13	0.22	0.88
Age (base = low)	−0.01	0.01	0.99	−0.01	0.01	0.99
Education Level (base = high)	0.26	0.17	1.29	0.09	0.15	1.09
Income (base = low)	0.04	0.05	1.04	0.00	0.05	1.00
**VARIABLES RELATED TO GAMBLING (STEP 2)**						
ERR_BEL (base = low)	0.14	0.12	1.15	0.40^***^	0.10	1.49
SYMB_M (base = low)	0.09	0.18	1.09	−0.21	0.16	0.81
ECON_M (base = low)	−0.23	0.18	0.80	−0.09	0.15	0.91
HEDO_M (base = low)	1.18^***^	0.22	3.26	0.35^*^	0.16	1.42
RISK (base = low)	0.25^*^	0.13	1.28	0.05^**^	0.01	1.05
IMPULS (base = low)	0.02	0.03	1.02	0.06^**^	0.02	1.06
FAMIL (base = no)	1.60^***^	0.45	4.96	1.47^***^	0.25	4.34
REG_SE (base = high)	1.26^***^	0.24	3.54	1.34^***^	0.18	3.82
AV_SE (base = high)	0.37	0.24	1.45	0.28	0.17	0.76
	**Step 1**			**Step 1**		
χ^2^(df)	9.82(4), *p* = 0.044			7.80(4), *p* = 0.099		
Hosmer and Lemershow test	χ^2^(*df* = 8) = 13.46, *p* = 0.097			χ^2^(*df* = 8) = 7.10, *p* = 0.525		
Nagelkerke R^2^	1.5%			1.2%		
Classification accuracy	94.9%			79.2%		
	**Step 2**			**Step 2**		
χ^2^(df)	416.26(13), *p* < 0.001			353.24(13), *p* < 0.001		
Hosmer and Lemershow test	χ^2^(df = 8) = 8.35, *p* = 0.400			χ^2^(df = 8) = 8.35, *p* = 0.400		
Nagelkerke R^2^	57.9%			46.5%		
Classification accuracy	96.4%			84.2%		

Dependent Variable, Gambling Severity Classification (0 = non-problematic gambler, 1 = at risk or problematic gamblers). B, Logistic regression coefficient; SE, Standard error of the logistic regression coefficient; OR, Odds Ratio; C.I., Confidence interval; ERR_BEL, Gamblers erroneous beliefs; SYMB_M, Symbolic motives for gambling; ECON_M, Economic motives for gambling; HEDO_M, Hedonic motives for gambling; RISK, Risk Taking; IMPULS, Impulsiveness; FAMIL, Having one or both parents who are or used to be excessive gamblers; REG_SE, Self-efficacy in self-regulating gaming behavior; AV_SE, Self-efficacy in avoiding gambling behavior;

* p < 0.05,

** p < 0.01 and

*** *p < 0.001*.

### Discussion

The positive psychometric properties of the MGSES were fully supported by results of this second study. The two-factor structure was replicated completely in two independent samples, and factorial invariance across two conditions was also supported. The 17-item MGSES proved to be a valid and reliable measure for the assessment of self-efficacy beliefs in the domain of gambling behavior.

Correlations and logistic regressions proved the criterion validity of the scale, demonstrating a coherent pattern of correlations with the criteria under study. Specifically, while both MGSES dimensions were negatively associated with various constructs and behaviors related to gambling, REG_SE—rather than AV_SE—was most significant in protecting an individual from excessive gambling behavior and, thus, resulted in a higher unique negative association with problem gambling indicators. This result appears particularly relevant considering the stress that has been placed, in the literature, on the ability to restrain or to avoid gambling. In fact, our results show clearly that it is not avoidance or a generic ability to control one's own behavior that really matters as a protective factor for problem gambling; what matters, rather, is the capability of self-regulation and self-control through specific gaming behaviors, such as spending only the amount of money that one initially decides to spend, ceasing to play when one's predetermined time limit has been reached, avoiding spending money on gambling that is needed for other expenses, and ceasing to play after losing a game. This result is further supported by the logistic regression analyses, which highlighted the fact that REG_SE additionally contributed to explaining the dependent variable of problem gambling (obtained by a combination of SOGS and PGSI) above and beyond the impact of all other variables.

In line with our expectations and with the literature (e.g., Bandura, 2007), REG_SE has proven to be particularly relevant when considering problem gambling, as suggested by odds ratios. The more gamblers perceive themselves as capable of controlling their gambling behavior, the less they exhibit behaviors that are indicative of excessive problem gambling. The greater explicative power of the REG_SE over AV_SE is in line with Bandura's theorization (Bandura, 1997).

## General discussion

This paper presented two independent studies, the results of which showed a clear convergence in supporting the psychometric properties of a new measure of self-efficacy within the domain of problem gambling: the MGSEG. An analysis of the literature on the role of self-efficacy in relation to gambling and gambling problems indicated that, although several studies have contributed to our knowledge of the role of self-efficacy in relation to gambling, these contributions were focused on the perceived ability to avoid gambling or restrain oneself from it. The self-regulation of gambling behavior was largely unexplored. The new self-efficacy scale, whose properties and characteristics are discussed here, provides a contribution aimed at filling this gap.

Through four different factor analyses on four independent samples of gamblers, the bi-dimensionality of the scale was demonstrated. The analyses supported the two posited dimensions and provided evidence for their high internal coherence. These dimensions refer to gamblers' beliefs about their capabilities regarding two factors: (a) self-regulating gambling behavior (REG_SE) and (b) avoiding risky gambling behavior (AV_SE). The importance of distinguishing between these two dimensions is attested by the findings from partial correlations with different measures of individual differences related directly or indirectly to gambling and with different behaviors regarded as markers of excessive gambling. Based on logistic regression, the two self-efficacy factors were considered, along with other factors, as independent variables, and problem gambling was considered as a dependent variable. In these analyses, the REG_SE dimension demonstrated a greater explicative power than the AV_SE dimension; moreover, REG_SE proved to be the stronger variable in explaining problem gambling, with the sole exception of familiarity.

This result is in line with SCT, which emphasizes the role of self-regulatory processes in the execution and modulation of behavior oriented toward the avoidance of negative consequences (e.g., Bandura, 1997, 2007). Gamblers who perceive themselves as more capable of regulating their own gaming behavior in order to realize a *positive* approach to gambling engage less frequently in excessive gambling. They spend less money for gamble, dedicate less time to gambling, and are less likely to be problem gamblers. Although the two factors of self-efficacy are highly correlated, they are also different. Their discriminant validity is not only proved by CFA, where the model assuming a single factor underlying the 17 items resulted in a much worse fit with respect to the two-factor target model, but also by the results of partial correlation and regression analyses. It is indeed important to measure these two different aspects of self-efficacy with respect to gambling: while the avoidance factor may plausibly be important and relevant in the evaluation of effectiveness of treatment, the regulative factor proves to be relevant (as evidenced by our findings) as far as responsible gaming behavior is concerned, and then mostly in a prevention context. Certainly, while the two factors are rooted in common self-regulative processes, they are not reducible to a single and general dimension.

### Limitations and future studies

Since the data used in the studies came from four representative samples, we must acknowledge that these data are of a cross-sectional nature. Accordingly, any claim regarding the predictive value of the self-efficacy dimensions must be made with great caution. Future longitudinal studies may address more solidly the paths of association among self-efficacy and the indicators of gambling behavior and problem gambling, as well as the test-retest reliability of the two scales. The studies used in this paper were conducted in a single country, and this may affect the generalizability of the results to other cultural contexts. Again, future studies are needed to further investigate both the psychometric properties of the scales as well as their correlation with problem gambling in national contexts other than Italy.

### Practical implications

The results from the two studies discussed here suggest some practical implications. The bi-faceted structure of MGSES appears consistent with a view of excessive gambling that is not limited to a focus on avoidance but, rather, takes into account other capabilities more relevant to *positive* gambling. As noted above, the MGSES may be used for a variety of purposes. In the prevention of excessive gambling and in the promotion of responsible gambling, the REG_SE scale may provide a useful tool for gathering relevant information that a gambler may use in self-assessment of his or her own gaming behavior in order to understand how this behavior may be adjusted or modulated to avoid the occurrence of excessive and unregulated gambling. For this purpose, it would be particularly useful to integrate this scale with measures of problem gambling (such as the short PGSI scale) that may allow evaluation, in a broader sense, of whether gamblers perceive themselves as able to manage their gambling behavior. The AV_SE scale may prove useful in assessing the progress of the problematic or pathological gambler in the various stages of the treatment process for pathological/excessive gambling as well as his or her ability to avoid relapses. In this regard, it would be useful to complement this scale with measures of self-efficacy related to other domains wherein the individual may exert his/her agency, such as self-regulation of emotions and self-regulation in resisting peer pressure to engage in harmful behaviors. Individuals' capability in these domains is crucial in fostering adaptive behaviors and avoiding maladaptive behaviors (Bandura et al., 2003). Moreover, in their recent review of the literature on self-efficacy in the treatment of substance-use disorders, Kadden and Litt (2011) not only indicated the positive relations among self-efficacy and treatment outcomes but claimed that effective treatment should improve patients' capacity to recognize their improved ability to cope with situations that present temptation to indulge in the addictive behavior at hand. The use of MGSES could be particularly useful in this regard for delivering feedback regarding patients' performance (in controlling and/or avoiding their gambling behavior) and comparing it with their past performance, both in real-life situations and in skill-training homework practice exercises as defined by the therapist.

## Author contributions

CB contributed on all sections of the paper. In particular he took care of the theoretical framework, to the literature review, to the methods, to discussion and conclusion, and to the definition of the data analytical strategy. VG contribute mainly to the data analysis, and to the writing of the results section. MV and RF contribute added their contribution to the writing of the methods, the results and the discussion sections.

### Conflict of interest statement

The funders (Lottomatica and Sisal) had no say in the analysis or report writing and agreed to allow publication at the Author's sole discretion.

## Footnotes

^1^As far as the check of the quality of the data is concerned, different analyses have been performed prior to substantial data analyses. In particular, the analysis of the pattern of missing values revealed that about 98% of the overall sample presented a percentage of missing value lower than 5%. In the online sample, no missing values were appreciated. Since a social desirability scale (composed by items from the Lie Scale of the Big Five Questionnaire, the personality questionnaire most used in Italy on adult samples) was administered in both samples, we also investigated the possibility of the prevalence of social desirable or of socially undesirable responses. In both sample the distribution of this scale scores was fairly normal (with kurtosis and skewness close to 0). Moreover, no significant differences emerged in this scale when problematic and non-problematic gamblers (classified using SOGS and PGSI) were compared.

^2^As in study 1 a check of the quality of the data was conducted prior to substantial data analyses. Results confirmed what emerged in study 1.

## References

[B1] American Psychological Society (2015). Replication in psychological science. Psychol. Sci. 26, 1827–1832. 10.1177/0956797615616374 26553013

[B2] Annis H. M. Graham J. M. (1988). Situational Confidence Questionnaire (SCQ) User's Guide. Toronto, ON: Addiction Research Foundation.

[B3] Bandura A. (1986). Social Foundations of Thought and Action: A Social Cognitive Theory. New York, NY: Prentice-Hall, Inc.

[B4] Bandura A. (1991). Social cognitive theory of self-regulation. Organ. Behav. Hum. Decis. Process. 50, 248–287.

[B5] Bandura A. (1997). Self-Efficacy: The Exercise of Control. New York, NY: Freeman

[B6] Bandura A. (2007). Much ado over faulty conception of perceived self-efficacy grounded in faulty experimentation. J. Soc. Clin. Psychol. 26, 641–758. 10.1521/jscp.2007.26.6.641

[B7] Bandura A. Locke E. A. (2003). Negative self-efficacy and goal effects revisited. J. Appl. Psychol. 88, 87–99. 10.1037/0021-9010.88.1.87 12675397

[B8] Bandura A. Caprara G. V. Barbaranelli C. Gerbino M. Pastorelli C. (2003). Role of affective self-regulatory efficacy in diverse spheres of psychosocial functioning. Child Dev. 74, 769–782. 10.1111/1467-8624.00567 12795389

[B9] Barbaranelli C. Vecchione M. Fida R. Podio-Guidugli S. (2013). Estimating the prevalence of adult problem gambling in Italy with SOGS and PGSI. J. Gambling Issues 28, 1–24. 10.4309/jgi.2013.28.3

[B10] Bentler P. M. (1990). Comparative fit indexes in structural models. Psychol. Bull. 107, 238–246. 10.1037/0033-2909.107.2.238 2320703

[B11] Blaszczynski A. Ladouceur R. Shaffer H. J. (2004). A science-based framework for responsible gambling: the Reno model. J. Gambling Stud. 20, 301–317. 10.1023/B:JOGS.0000040281.49444.e2 15353926

[B12] Byrne B. M. (2010). Structural Equation Modeling with Mplus: Basic Concepts, Applications, and Programming, 2nd Edn. New York, NY: Routledge.

[B13] Casey L. M. Oei T. P. S. Melville K. M. Bourke E. Newcombe P. A. (2008). Measuring self-efficacy in gambling: the Gambling Refusal Self-Efficacy Questionnaire. J. Gambling Stud. 24, 229–246. 10.1007/s10899-007-9076-2 17849178

[B14] Cheung G. W. Rensvold R. B. (2002). Evaluating goodness-of-fit indexes for testing measurement invariance. Struct. Equat. Model. 9, 233–255. 10.1207/S15328007SEM0902_5

[B15] Cotte J. (1997). Chances trances and lots of slots. Gambling motives. J. Leisure Res. 29, 380–407.

[B16] Dahlbäck O. (1990). Personality and risk-taking. Pers. Individ. Dif. 11, 1235–1242. 10.1016/0191-8869(90)90150-P

[B17] DeShon R. P. (2004). Measures are not invariant across groups with error variance homogeneity. Psychol. Sci. 46, 137–149.

[B18] DiClemente C. C. Fairhurst S. K. Piotrowski N. A. (1995). Self-efficacy and addictive behaviors, in Self-Efficacy, Adaptation, and Adjustment: Theory Research, and Application, ed Maddux J. E. (New York, NY: Plenum Press), 109–138.

[B19] Ferris J. Wynne H. (2001). The Canadian Problem Gambling Index: User Manual. Ottawa, ON: Canadian Centre on Substance Abuse.

[B20] Hayton J. C. Allen D. G. Scarpello V. (2004). Factor retention decisions in exploratory factor analysis: a tutorial on parallel analysis. Organ. Res. Methods 7, 191–205. 10.1177/1094428104263675

[B21] Hu L. T. Bentler P. M. (1999). Cutoff criteria for fit indexes in covariance structure analysis: conventional criteria versus new alternatives. Struct. Equat. Model. 6, 1–55. 10.1080/10705519909540118

[B22] Judge T. A. Jackson C. L. Shaw J. C. Scott B. A. Rich B. L. (2007). Self-efficacy and work-related performance: the integral role of individual differences. J. Appl. Psychol. 92, 107–127. 10.1037/0021-9010.92.1.107 17227155

[B23] Kadden R. M. Litt M. D. (2011). The role of self-efficacy in the treatment of substance use disorders. Addict. Behav. 36, 1120–1126. 10.1016/j.addbeh.2011.07.032 21849232 PMC3179802

[B24] Karademas E. C. (2006). Self-efficacy, social support and well-being: the mediating role of optimism. Pers. Individ. Dif. 40, 1281–1290. 10.1016/j.paid.2005.10.019

[B25] Kline R. B. (2016). Principles and Practice of Structural Equation Modeling, 4th Edn. New York, NY: Guilford Press.

[B26] Lesieur H. R. Blume S. B. (1987). The South Oaks Gambling Screen (SOGS): a new instrument for the identification of pathological gamblers. Am. J. Psychiatry 144, 1184–1188. 10.1176/ajp.144.9.1184 3631315

[B27] MacCallum R. C. Browne M. W. Sugawara H. M. (1996). Power analysis and determination of sample size for covariance structure modeling. Psychol. Methods 1, 130–149. 10.1037/1082-989X.1.2.130

[B28] Marlatt G. A. (1985). Situational determinants of relapse and skill training interventions, in Relapse Prevention: Maintenance Strategies in Treatment of Addictive Behaviours, eds Marlatt G. A. Gordon J. R. (New York, NY: The Guilford Press), 71–127.

[B29] May R. K. Whelan J. P. Steenbergh T. A. Meyers A. W. (2003). The gambling self-efficacy questionnaire: an initial psychometric evaluation. J. Gambling Stud. 4, 339–357. 10.1023/A:1026379125116 14634297

[B30] Meredith W. (1993). Measurement invariance, factor analysis and factorial invariance. Psychometrika 58, 525–543. 10.1007/BF02294825

[B31] Moritz S. E. Feltz D. L. Fahrbach K. R. Mack D. E. (2000). The relation of self-efficacy measures to sport performance: a meta-analytic review. Res. Q. Exerc. Sport 71, 280–294. 10.1080/02701367.2000.10608908 10999265

[B32] Muthén L. K. Muthén B. O. (1998-2016). Mplus User's Guide, 7th Edn. Los Angeles, CA: Muthén & Muthén.

[B33] Pajares F. Urdan T. C. (2006). Self-Efficacy Beliefs of Adolescents. Greenwich, CT: Information Age Publishing.

[B34] Patton J. H. Stanford M. S. Barratt E. S. (1995). Factor structure of the Barratt Impulsiveness Scale. J. Clin. Psychol. 51, 768–774. 10.1002/1097-4679(199511)51:6<768::AID-JCLP2270510607>3.0.CO;2-1 8778124

[B35] Rousseau G. G. Venter D. J. L. (2002). Measuring consumer attitudes towards gambling. SA J. Indus. Psychol. 28, 87–92. 10.4102/sajip.v28i2.50

[B36] Schuman H. Presser S. (1996). Questions and Answers in Attitude Surveys: Experiments on Question Form, Wording, and Context. Thousand Oaks, CA: Sage.

[B37] Steenbergh T. Meyers A. May R. Whelan J. (2002). Development and validation of the Gamblers' Beliefs Questionnaire. Psycholo. Addict. Behav. 16, 143–149. 10.1037//0893-164X.16.2.143 12079253

[B38] Steiger J. H. (1990). Structural model evaluation and modification: an interval estimation approach. Multivariate Behav. Res. 25, 173–180. 10.1207/s15327906mbr2502_4 26794479

[B39] Strobel M. Tumasjan A. Spörrle M. (2011). Be yourself, believe in yourself, and be happy: self-efficacy as a mediator between personality factors and subjective well-being. Scand. J. Psychol. 52, 43–48. 10.1111/j.1467-9450.2010.00826.x 20497398

[B40] Tabachnick B. G. Fidell L. S. (2007). Using Multivariate Statistics, 5th Edn. Boston, MA: Pearson Education, Inc.

[B41] Tangney J. P. Baumeister R. F. Boone A. L. (2004). High self-control predicts good adjustment, less pathology, better grades, and interpersonal success. J. Pers. 72, 271–324. 10.1111/j.0022-3506.2004.00263.x 15016066

[B42] Winfree W. R. Ginley M. K. Whelan J. P. Meyers A. W. (2014). Psychometric evaluation of the Gambling Self-Efficacy Questionnaire with treatment-seeking pathological gamblers. Psychol. Addict. Behav. 28, 1305–1310. 10.1037/a0037678 25180557

[B43] Wood R. T. Griffiths M. D. (2015). Understanding positive play: an exploration of playing experiences responsible gambling practices. J. Gambling Stud. 31, 1715–1734. 10.1007/s10899-014-9489-7 25209455

[B44] Young R. M. Oei T. P. S. (1996). Drinking Expectancy Profile: Test Manual. Behaviour Research and Therapy Centre; University of Queensland.

[B45] Young R. M. Oei T. P. S. Crook G. M. (1991). Development of a drinking self-efficacy questionnaire. J. Psychopathol. Behav. Assess. 13, 1–15. 10.1007/BF00960735

[B46] Zaleskiewicz T. (2001). Beyond risk seeking and risk aversion: personality and the dual nature of economic risk taking. Eur. J. Pers. 15, S105–S122. 10.1002/per.426

