# Supplementary material for: Psychometric Characteristics of a New Scale for Measuring Self-efficacy in the Regulation of Gambling Behavior
Source: Front Psychol. 2017 Jun 20;8:1025. doi: 10.3389/fpsyg.2017.01025 (PMC5477641; doi:10.3389/fpsyg.2017.01025)
Supplement: Supplementary file 1 [file DataSheet1.docx]

Appendix

*Items of the Multidimensional Gambling Self-Efficacy Scale* (MGSES)

*Self-efficacy in self-regulating gaming behavior Subscale*

***How are you capable to***

it1. Play only the amount of money that you initially decided to play, without going further

it2. Stop to play when you went over the time limit you initially defined to dedicate to play

it3. Avoid to spend in games money that must spent to buy other things

it4. Maintain the decision not to play when you decided so

it5. Avoid to spend in games the change

it6. Stop to play when you loose and you want to continue to play to win it back

*Self-efficacy in avoiding gaming behavior* *Subscale*

***How much are you capable to avoid to play***

it7. When you are under pressure (at work at school) or you have worries and personal problems

it8. When you are depressed, tense or anxious

it9. When you are in vacations or in your spare time

it10. When you are alone

it11. When there are other interesting activities the you could do

it12. When you are with your friends

it13. When you feel bored

it14. When you had an argument with a member of your family or with one friend of yours

it15. When you are relaxed and you want to have fun

it16. When you feel the urge to play

it17. When you feel physical discomfort (e.g., you have pain in the stomach or difficulty in sleeping) because you did not played since some time

*Response format: 1= not at all, 2= Few, 3 = moderately; 4 = a lot; 5 = Completely)*
